# MicroRNA analysis of medium/large placenta extracellular vesicles in normal and preeclampsia pregnancies

**DOI:** 10.3389/fcvm.2024.1371168

**Published:** 2024-04-02

**Authors:** Toluwalase Awoyemi, Shuhan Jiang, Maryam Rahbar, Prasanna Logentherian, Gavin Collett, Wei Zhang, Adam Cribbs, Sofia Cerdeira, Manu Vatish

**Affiliations:** ^1^ Nuffield Department of Women’s & Reproductive Health, University of Oxford, Oxford, United Kingdom; ^2^ Nuffield Department of Orthopaedics, Rheumatology and Musculoskeletal Sciences, University of Oxford, Oxford, United Kingdom

**Keywords:** syncytiotrophoblast membrane extracellular vesicles (STB-EVs), preeclampsia, biomarkers, microRNA, placenta EVs, mechanisms

## Abstract

**Background:**

Preeclampsia (PE) is a hypertensive disorder of pregnancy, affecting 2%–8% of pregnancies worldwide, and is the leading cause of adverse maternal and fetal outcomes. The disease is characterized by oxidative and cellular stress and widespread endothelial dysfunction. While the precise mechanisms are not entirely understood, the pathogenesis of PE is closely linked to placental dysfunction and, to some extent, syncytiotrophoblast extracellular vesicle release (STB-EVs). These vesicles can be divided into the less well-studied medium/large EVs (220–1,000 nm) released in response to stress and small EVs (<220 nm) released as a component of intercellular communication. The previously described production of m/lSTB-EVs in response to cellular stress combined with the overwhelming occurrence of cellular and oxidative stress in PE prompted us to evaluate the microRNAome of PE m/lSTB-EVs. We hypothesized that the microRNAome profile of m/lSTB-EVs is different in PE compared to normal pregnancy (NP), which might permit the identification of potential circulating biomarkers not previously described in PE.

**Methods/study design:**

We performed small RNA sequencing on medium/large STB-EVs isolated from PE and NP placentae using dual-lobe ex vivo perfusion. The sequencing data was bioinformatically analyzed to identify differentially regulated microRNAs. Identified microRNAs were validated with quantitative PCR analysis. We completed our analysis by performing an in-silico prediction of STB-EV mechanistic pathways.

**Results:**

We identified significant differences between PE and NP in the STB-EVs micro ribonucleic acid (microRNA) profiles. We verified the differential expression of *hsa-miR-193b-5p, hsa-miR-324-5p, hsa-miR-652-3p, hsa-miR-3196, hsa-miR-9-5p, hsa-miR-421, and hsa-miR-210-3p in the medium/large STB-EVs.* We also confirmed the differential abundance of *hsa-miR-9-5p* in maternal serum extracellular vesicles (S EVs)*.* In addition, we integrated the results of these microRNAs into the previously published messenger RNA (mRNA) data to better understand the relationship between these biomolecules.

**Conclusions:**

We identified a differentially regulated micro-RNA, *hsa-miR-9-5p,* that may have biomarker potential and uncovered mechanistic pathways that may be important in the pathophysiology of PE.

## Introduction

Preeclampsia (PE) is a multisystemic disorder of pregnancy characterized by hypertension (systolic blood pressure ≥140 mmHg/diastolic pressure ≥90 mmHg) and either proteinuria (protein/creatinine ratio of 0.3 mg/mmol or more) or evidence of maternal acute kidney injury, neurological features, and/or fetal growth restriction and in severe cases, death (1). The diagnosis of PE remains challenging, and the mechanism of the disease remains elusive (2, 3) PE is a disease characterized by syncytiotrophoblast stress (4). The placenta is integral to its pathogenesis, releasing soluble factors and extracellular vesicles into the maternal circulation (5). These placenta-derived EVs, termed syncytiotrophoblast membrane extracellular vesicles (STB-EVs), express various biomolecules such as proteins, messenger RNA (mRNA), micro-RNA, and transfer RNA (tRNA) in their cargo (6, 7). These EVs include the more studied, small STB-EVs (with a size range of less than 220 nm) and the less well-studied medium/large STB-EVs (221 nm–1,000 nm). The biogenesis of EVs may be more relevant, given that medium/large EVs are released because of stress, while small EVs appear to be constitutively released (4).

MicroRNAs are a sub-class of small non-coding RNAs comprising 19–24 nucleotides. Micro-RNAs regulate mRNA by inducing mRNA cleavage or repressing translation (8, 9) In the circulation, micro-RNAs can be carried by extracellular vesicles (EVs), argonaut 2 (AGO2) proteins, high-density lipoprotein, and other ribonucleoprotein complexes and circulate freely in the blood (10). In Preeclampsia, the abnormal microRNA expression patterns in the circulation or placenta have been linked to the pathology. These expression changes may influence the immune functions, angiogenesis, trophoblast proliferation, and invasion seen in PE. The stressed preeclampsia placenta may release EVs carrying signals that impact maternal systems (4). Circulating microRNAs have primarily been explored in PE by interrogating cell-free microRNA in the circulation, identifying potential biomarkers, and then inferring these to the placenta. Srinivasan et al. and Sheikh et al. recently summarised most previously identified preeclamptic extracellular microRNA biomarker candidates (11, 12). In addition, Srinivasan et al. also suggested new microRNAs such as hsa-miR 941, hsa-miR-516b-5p, hsa-miR-let-7b-5p/hsa-miR-155-5p as being of placental origin. Still, no attention was paid to which subspecies of placenta EVs express these microRNAs, i.e., whether small or medium/large STB-EVs (11). In contrast, Chamley et al. approached this question using placenta EVs or debris derived from placenta explants (12). However, using explants rather than ex-vivo dual lobe placenta perfusion possibly does not enable full exploration of the interactions occurring within placenta components and cells *in vivo*.

Srinivasan et al. used pooled serum followed by immunoprecipitation of extracellular markers with the pan EV marker CD63, placenta EVs with PLAP, and anti-AGO2 (11). In contrast, Wei et al. explored the role of placenta debris in PE and discovered that PE placenta debris contains a microRNA profile different from that of normal pregnancy (13). Their EVs were generated with placental explants, a less physiological model than ex-vivo dual lobe perfusion (14). However, the small RNA composition of the EVs from the explant culture differs from that derived from the ex-vivo dual lobe perfusion. Chamley et al. reported that most of their EVs generated from placenta explants are made up of microRNAs.

In contrast, most of the small RNA sequences generated from ex-vivo dual lobe perfusion comprise tRNA half fragments (15) Finally, most researchers have focused on exosomes, small STB-EVs, or whole serum samples rather than medium/large STB-EVs as biomarkers. We instead concentrate on medium/large STB-EVs in normal and PE placentae that had been isolated from the source organ using dual lobe ex vivo placental perfusion and serum EVs, given that medium/large STB-EVs are released in response to stress, and Preeclampsia is a stressful condition.

We hypothesized that PE m/lSTB-EVs contain a different microRNA profile than NP, which may offer potential diagnostic and mechanistic insights. We also correlated our microRNA analysis results with our previously described messenger RNA (mRNA) analysis from the same population (16). By correlating the microRNA and mRNA profiles, we aim to enhance our understanding of the molecular mechanisms underlying Preeclampsia and pave the way for developing novel diagnostic and therapeutic strategies.

## Materials and methods

The primary resource table and research resource identifiers (RRID) used in this paper can be found in the Supplementary Material.

### Ethics approval and patient information

We obtained approval for this study from the Oxfordshire Research Ethics Committee C (Reference Number: 07/H0606/148). We defined normal pregnancy as a healthy singleton pregnancy conceived naturally without known fetal abnormality or abnormal maternal blood pressures. Placentas were collected from normal pregnant women who were in labor and undergoing elective Caesarean sections. Serum samples were obtained from gestationally matched PE and NP patients. We defined PE as hypertension (systolic blood pressure ≥140 mmHg/diastolic pressure ≥90 mmHg) and proteinuria (protein/creatinine ratio of 0.3 mg/mmol or more). In addition, for the serum samples, we selected EOPE patients with a soluble Flt: PLGF (sFLT: PLGF) ratio >110 and the NP samples with a ratio <38. The soluble Flt: PLGF (sFLT: PLGF) ratio helps characterize the level of placenta ischemia in Preeclampsia and is now routinely used in risk-stratifying Preeclampsia (17) We obtained informed consent from all participants in this study.

### Sample preparation and enrichment of syncytiotrophoblast membrane extracellular vesicles (STB-EVs)

We enriched for m/lSTB-EVs as described in our previous study (18). Briefly, after examining the placenta, a satisfactory cotyledon of the placenta devoid of rupture, ischemia, clots, or calcification was identified. This cotyledon was perfused at a flow rate of 4–5 ml/min for three hours to obtain a maternal placenta perfusate using dual lobe perfusion. STB-EVs were enriched by differentially centrifuging the maternal placental perfusate; an initial 1,500 g spin at 4°C (*Beckman Coulter Avanti J-20XP centrifuge using a Beckman Coulter JS-5.3 swing-out rotor*) to remove red blood cells and cellular debris followed by enrichment for m/lSTB-EVs by centrifugation at 10,000 × g in a swing bucket centrifuge (*Beckman L80 ultracentrifuge and Sorvall TST28.39 swing-out rotor*) for 30 min at 4°C. Pellets were resuspended in filtered phosphate-buffered saline (fPBS, 0.22 µm Millipore Stericup Filtration Device (Merck, SCVPU11RE) and aliquots with a protein concentration around ≈ 5 µg/µl (measured using a Pierce BCA protein assay) were stored or set aside for downstream characterization. Downstream characterization experiments included nanoparticle tracking analysis, transmission electron microscopy, western blot, and flow cytometry. The STB-EV enrichment and categorization process has been deposited and assessed on EV Track [(http://www.EVTRACK.org), EV-TRACK ID: EV210382] with a score of 78% (the average score on EV track for 2021 is 52%). For the serum validation, a cohort of serum samples from pregnant women with early onset preeclampsia (EOPE, *n* = 8) and gestationally matched normal pregnancy women (NP) were obtained (*n* = 8). The serum samples were prepared using standard protocol. Briefly, blood samples were collected from the antecubital fossa and centrifuged at 2,000 g for 10 min. The supernatant was collected, and the serum samples were frozen at −80°C until analysis. All blood samples were processed within 1 h upon collection.

To prepare the placenta tissue for RNA extraction, placental tissue samples were promptly collected post-delivery and precisely sliced before being immersed in pre-cooled homogenization vessels containing 500 µl of RNAprotect Tissue Reagent. Ensuring RNA stability, these prepared samples were swiftly stored at −80°C. For RNA extraction, the RNAprotect-treated tissue was transferred to appropriately sized vessels, and 600 µl of Buffer RLT was added. Under ice-cooled conditions, the samples underwent thorough homogenization for 40 s with a tissue homogenizer, resulting in a consistent lysate. The lysate was centrifuged at full speed for 3 min, yielding an RNA-enriched supernatant. To prevent contamination, the supernatant was meticulously transferred to fresh RNase-free tubes. The RNA lysates were then securely stored at −80°C until further RNA extraction procedures were performed following the standard manufacturer protocol with the RNeasy Mini kit. Stringent measures, including the use of RNase-free equipment, were consistently applied throughout the process to mitigate the risk of RNA degradation.

### Characterization of syncytiotrophoblast membrane extracellular vesicles (STB-EVs) enrichment with flow cytometry, transmission electron microscopy (TEM), western blot (WB), and nanoparticle tracking analysis (NTA)

We phenotyped the isolated m/lSTB-EV with flow cytometry (BD Biosciences, LSRII), transmission electron microscopy (TEM, for morphology), and western blot (for immunophenotyping). We used antibodies to placental alkaline phosphatase—PLAP- (to confirm syncytiotrophoblast origin), CD41 (to identify co-isolated platelet EVs, CD235a/b (to identify co-isolated red blood cell EVs), HLA class I and II (to identify co-isolated white blood cell EVs) for flow cytometric analysis as previously described in our previous paper (16). For immunophenotyping, we characterized the m/lSTB-EVs by probing for known EV markers CD63 [(200 mg/ml) at 1:1,000 dilution, Sc-59286, Santa Cruz Biotechnology], placental alkaline phosphatase [PLAP (1.667 mg/ml) at 1:1,000 dilution, in house antibody], and the known negative EV marker Cytochrome C [(200 mg/ml) at 1:1,000 dilution, Sc-13156, Santa Cruz Biotechnology] as recommended by the international society for extracellular vesicles (ISEV) (19). The immunoblot was subsequently incubated with either anti-mouse or anti-rabbit polyclonal goat immunoglobulins/HRP (at 1 2,000 dilution, Dako UK Ltd, Cambridgeshire UK).

Morphology was assessed by using TEM. Briefly, STB-EVs were diluted with filtered PBS to obtain a protein concentration between 0.1–0.3 µg/µl. 10 µl of the solution was added to freshly glowing discharged carbon formvar 300 mesh copper grids for two minutes, dried with filter paper, stained with 2% uranyl acetate for ten seconds, and air-dried. The grid was negatively stained to intensify the contrast between the pellets and the background. Imaging was done with an FEI Tecnai 12 TEM at 120 kV with a Gatan OneView CMOS camera.

Finally, we characterized the STB-EVs with nanoparticle tracking analysis ((NTA) NanoSight NS500 instrument equipped with a 405 nm laser (Malvern UK), sCMOS camera, and NTA software version 3.5, Build 0033 (Malvern UK)). We checked the instrument performance with silica 100 nm microspheres (Polysciences, Inc.). Each STB-EV sample was diluted in fPBS to obtain the concentration within 2 × 10^2^ particles/ml to 1 × 10^9^ particles/ml. The samples were automatically injected into the sample chamber with a 1 ml syringe with the following script for EV measurements: PRIME, DELAY 5, CAPTURE 60, REPEAT 4. Images of the analyzed samples were captured on camera at 12 (Camera shutter speed; 15 ms and Camera gain; 350), and NTA post-acquisition settings were optimized and kept constant between samples. Each video recording was analyzed to infer STB-EV size and concentration profile.

### Library preparation, sequencing, and bioinformatic analysis of small RNA sequences

For sequencing, RNA extraction and sample preparations were performed for both placenta lysates and medium/large STB-EVs (6 NP, 6 PE) with the RNeasy Mini kit and miRCURY™ RNA isolation kit for biofluids (Exiqon Services, Denmark) respectively based on the manufacturer's protocol. RNA purity was assessed with Nanodrop, and samples with a 260/280 ratio greater than 1.8 were sent to the Welcome Centre for Human Genetics (WCHG) for sequencing. RNA libraries were prepared using the standard procedure using the TruSeq small RNA library preparation kits (Illumina). Details can be found in the Supplementary Data.

We performed bioinformatics analysis for small RNA analysis on Oasis 2.0 (https://oasis.ims.bio/small_rna_classification.php) (20) using the default small RNA pipeline. More information about the default parameter setting for OASIS-db can be found in the documentation and the Supplementary Methods. Differential expression was performed with DESeq2 package (21) (v.1.32.0 in R v.4.0.5), and the Benjamin Hochberg method was used to correct for multiple testing and calculate the adjusted *P* value. Significantly differentially expressed microRNAs were defined by fold change (FC) ≥ ± 1 with an adjusted *P* value ≤ 0.05. The unprocessed fastQ files and the processed data have been archived in the National Center for Biotechnology Information (NCBI) database. These files are publicly accessible under the accession number GSE190972.

### Complementary cDNA synthesis for microRNA quantitative polymerase chain reaction (qPCR)

Complementary DNA (cDNA) from the extracted RNA was generated with the TaqMan^TM^ Advanced microRNA cDNA Synthesis Kit (A28007). Each reaction was performed in a G-storm^TM^ (G-storm, UK) thermocycler with settings provided by the manufacturer. We selected the following m/lSTB-EV microRNAs to validate based on fold change in PE compared to NP; hsa*-miR-30b-5p, hsa-miR-221-3p, hsa-miR-222-3p, hsa-miR-324-5p, hsa-miR-194-5p, hsa-miR-519c-3p, hsa-miR-652-3p, hsa-miR-584-5p, hsa-miR-421, hsa-let-7e-5p, hsa-miR-3196, hsa-miR-4488, hsa-miR-4516, hsa-miR-9-5p, hsa-miR-210-3p, and hsa-miR-193b-5p* (see Supplementary Material). We opted for hsa-miR-9-5p for serum validation *due to its described role in angiogenesis, a process widely reported dysregulated in Preeclampsia.* All microRNAs tested were detected before the 35th cycle. The following settings were used for the QuantStudio 3 and QuantStudio Design and Analysis desktop software (Thermofisher) qPCR: hold 50°C for 2 min, hold at 95°C for 20 s, followed by forty 95°C for 1 s and 60°C for 20 s.

For placenta and m/lSTB-EVs, data was normalized to *hsa-miR-30d-5p*. We chose hsa-miR-30d-5p from the sequencing data based on an absent differential expression (FC approximately <0.001) between PE and NP and a low standard error (LFSE) and verified by qPCR for expression stability. For serum samples, data was normalized to spiked in Cel_miR-39. Analysis was performed with the 2^−*ΔΔ*Ct^ method (22) and statistical testing on the *Δ* Ct values using a one-tailed Student *t-test (*significance = *P* < 0.05). Data was visualized using GraphPad Prism software (version 9) and expressed as fold change, and the standard error of the mean was denoted as error bars. All gene expression assays, their corresponding assay IDs, and all other details used in this study are listed in Supplementary Methods.

### RNA extraction, complementary cDNA synthesis, and qPCR for serum extracellular vesicle

Frozen serum samples from gestationally age-matched women with EOPE (*n* = 8) and those with normal pregnancies (*n* = 8) were thawed at 37°C in a water bath. Subsequently, 800 µl of serum was loaded onto ExoRneasy Midi kits (Qiagen) columns to extract high-quality RNA. We then added 1 μl aliquot of a 5 nanomolar working solution of cel-miR-39 (Integrated DNA Technologies, 100 nmoles RNA Oligo) spike-in to each serum sample. Complementary DNA (cDNA) synthesis was performed using the TaqMan^TM^ Advanced microRNA cDNA synthesis kit (A28007). All reactions were meticulously executed on ice, adhering to the manufacturer's protocol. Post reverse transcription (RT), 10 µl of the reverse-transcribed samples underwent pre-amplification. The pre-amplified samples were then diluted at a 1:5 ratio with 0.1× Tris-EDTA buffer for subsequent qPCR reactions, as earlier described.

### Functional enrichment analysis of microRNA analysis

Given our specialized interest in microRNAs and their regulatory role in transcription, a comprehensive functional enrichment analysis was conducted on microRNAs extracted from both placenta and m/lSTB-EVs. Utilizing DIANA-miRPath v3.0 (http://www.microrna.gr/miRPathv3), the microRNAs were stratified into upregulated and downregulated categories for analysis. We performed distinct functional enrichment analyses, focusing on Gene Ontology categories encompassing biological processes, molecular functions, cellular components, and Kyoto Encyclopedia of Genes and Genomes (KEGG) pathways. This analysis was conducted separately for the top 100 upregulated and downregulated microRNAs identified in the placenta and m/lSTB-EVs. Restricting the examination to up to 100 microRNAs (microRNAs) with a fold change (LFC ≥ 0.75) and an adjusted *p*-value (<0.05), the imposed limit was imposed by the default settings of the tool and the computational demands associated with processing extensive lists of microRNAs. The prediction of mRNA targets for the loaded microRNAs was accomplished using the microT-CDS algorithm with a microT-threshold of 0.8. To refine the results further, the outcomes of this analysis were fused at the level of categories or pathways. Additionally, removing, summarizing, and visualizing redundant Gene Ontology (GO) terms were undertaken using REVIGO (http://revigo.irb.hr/) to address the computational intensity and streamline the data.

### MicroRNA-messenger RNA correlation analysis

We subsequently focused on predicting and visualizing mRNA targets modulated by the differentially expressed microRNAs detected in our study to elucidate the regulatory dynamics associated with Preeclampsia. A vital part of this study was identifying some microRNAs responsible for the differentially expressed mRNAs previously reported in our transcriptomics paper (16). The raw fastQ files and the processed file of the transcriptomics data can be accessed on NCBI with the following ID: GSE190971. This analysis used the normalized expression matrices for microRNAs and mRNAs with miRTarVis + (http://sehilyi.com/mirtarvisplus/)-an online interactive visual analytics tool. MicroRNA expression matrices were filtered based on a fold change (FC) threshold of 1.5 and an adjusted *p*-value criterion of 0.05. Concurrently, mRNA expression data underwent a similar filtration process, with a fold change (FC) threshold of 1.5 and a stringent adjusted *p*-value of 0.005. The filtered datasets were harnessed to predicticroRNA target interactions with the TargetScan prediction algorithm (23). After making the prediction, we used Pearson correlation, mutual information, and maximal information-based nonparametric exploration (MINE) to find microRNA-mRNA pairs that were strongly correlated. We selected the top 300 interactions for the placenta and the top 1,000 interactions for m/lSTB-EVs. A correlation coefficient cutoff of 0.5 and a neighbor cutoff score of 3 were used for correlation and mutual information analysis. We only considered microRNA-mRNA interactions identified by all three algorithms for enhanced reliability. The results of the microRNA-mRNA interactions were visualized through a treemap, providing a comprehensive overview of the intricate regulatory network governing the interplay between microRNAs and mRNAs in Preeclampsia.

## Result

### Patient demographics and clinical characteristics of placental and m/lSTB-EV samples

PE women were (as expected) significantly more likely to deliver prematurely (PE = 31.88 weeks' gestation NP = 39.33 weeks' gestation) and have proteinuria (PE = 2.19 pluses on urine dipstick, NP = 0 pluses on urine dipstick) compared to normal pregnancy. PE mothers had a significantly higher average systolic (163.12 mmHg) and diastolic (104.62 mmHg) blood pressure compared to normal pregnant mothers average systolic (121.47 mmHg) and diastolic (71.2 mmHg). PE babies weighed less than normal babies (PE = 1,515.31 g, NP = 3,671.33 g). However, we found no significant difference in body mass index, the gender of the child, and maternal age (Table 1).

**Table 1:** General descriptive statistics of the sample population for medium/large (m/lSTB-EVs) study.

Characteristics	Normal pregnancy	Preeclampsia	*P* Value
Sample size	6	6	
Maternal age years [mean (SD)]	34.07 ± 5.42	34.12 ± 4.63	0.974
Systolic blood pressure mmHg [mean (SD)]	121.47 ± 9.49	163.12 ± 14.66	<0.001
Diastolic blood pressure mmHg [mean (SD)]	71.2 ± 5.56	104.62 ± 7.36	<0.001
Body mass index kg/m^−2^ [mean (SD)]	27.7 ± 7.52	29.09 ± 9.4	0.653
Proteinuria plus(es) [mean (SD)]	0	2.19 ± 1.15	<0.001
Gestational age at delivery in weeks [mean (SD)]	39.33 ± 0.72	31.88 ± 3.52	<0.001
Birth weight grams [mean (SD)]	3,671.33	1,515.31 ± 726.65	<0.001
Intrauterine growth restriction (IUGR) = Yes (%)	0 (0)	15 (93.8)	<0.001
Male new-born gender (%)	5 (33.3)	6 (37.5)	1

### Characterization of STB-EV by transmission electron microscopy (TEM), nanoparticle tracking analysis (NTA), western blot (WB)

**Figure 1:**
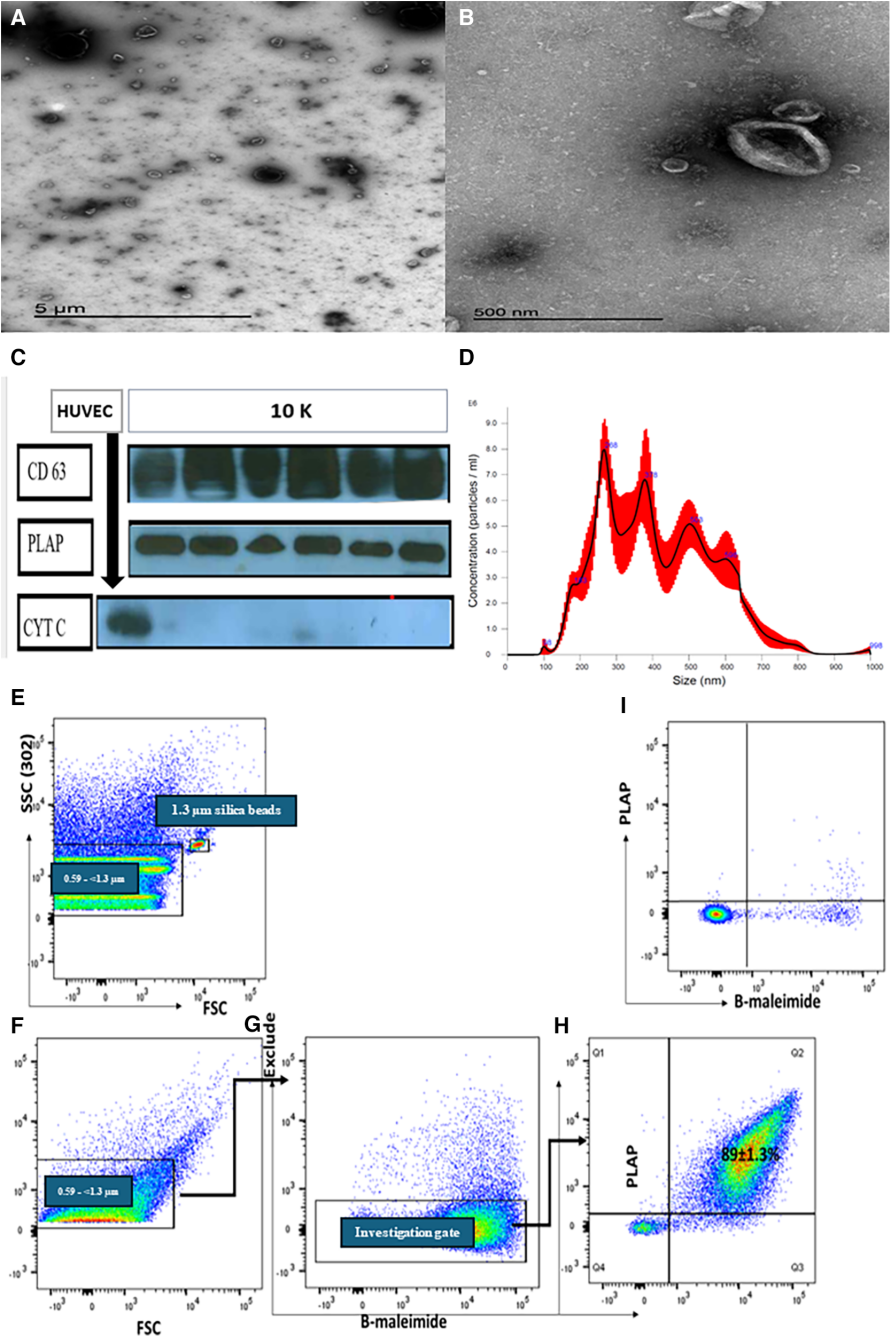
Representative transmission electron microscopy (TEM) images showing a wide angle (**A**) and narrow-angle (**B**) views of medium/large STB-EVs. (**C**) Western blot of m/lSTB-EVs (10 K) demonstrates PLAP positivity in all samples, confirming their placental origin. The absence of cytochrome C in the STB-EV population further attests to the absence of contamination. (**D**) Nanoparticle tracking analysis (NTA) results for m/lSTB-EVs reveal a broad size distribution. Panels (**E**–**I**) depict representative flow cytometric analysis of m/lSTB-EVs enriched by placental perfusion. Apogee beads mix was utilized to set the flow machine's light scatter resolution to 0.59–1.3 mm silica beads. Panels (**E**,**F**) illustrate the application of SSC and FSC PMTVs, determined by apogee beads mix to analyze m/lSTB-EVs. STB-EVs from the investigation gate were subsequently analyzed for PLAP expression and bioM staining [Panels (**G**,**H**)]. This analysis revealed a high percentage (89%) of PLAP-positive vesicles. These PLAP + bioM + EVs exhibited high sensitivity to detergent treatment, as evidenced by the reduction in PLAP + bioM ++ in Panel I, confirming their vesicular nature.

The 10 K STB-EVs isolated from placenta perfusion showed the characteristic cup shape under the negative staining on transmission electron microscopy (TEM). Western blot confirmed that 10 K STB-EVs expressed the classic syncytiotrophoblast membrane marker (CD63), placenta alkaline phosphatase (PLAP), confirming placental origin (Figure 1C). In addition, 10 K STB-EVs lacked the negative EV marker cytochrome C, confirming that these vesicles were not derived from apoptotic bodies (Figure 1C). Nanoparticle tracking analysis (NTA) confirmed the heterogeneity of the 10 K STB-EVs (Figure 1D) with a size range of (479.4 ± 145.6) nm, with the majority over 200 nm. According to ISEV, 10 K STB-EVs were defined as medium/large STB-EVs (m/lSTB-EVs). Finally, flow cytometric analysis (Figures 1E–I) of m/l STB-EVs showed PLAP positive 89 ± 1.3% predominantly. m/l STB-EVs were also shown to be vesicular due to minimal signal after adding detergent to break the lipid membrane (Figure 1I). These data suggested that we had successfully enriched m/lSTB-EVs from placental perfusions. Full details of m/lSTB-EV characterization can be seen in the Supplementary Methods.

### Identification and validation of differentially expressed microRNAs (DEMs) in STB-EVs and maternal serum EVs

**Figure 2:**
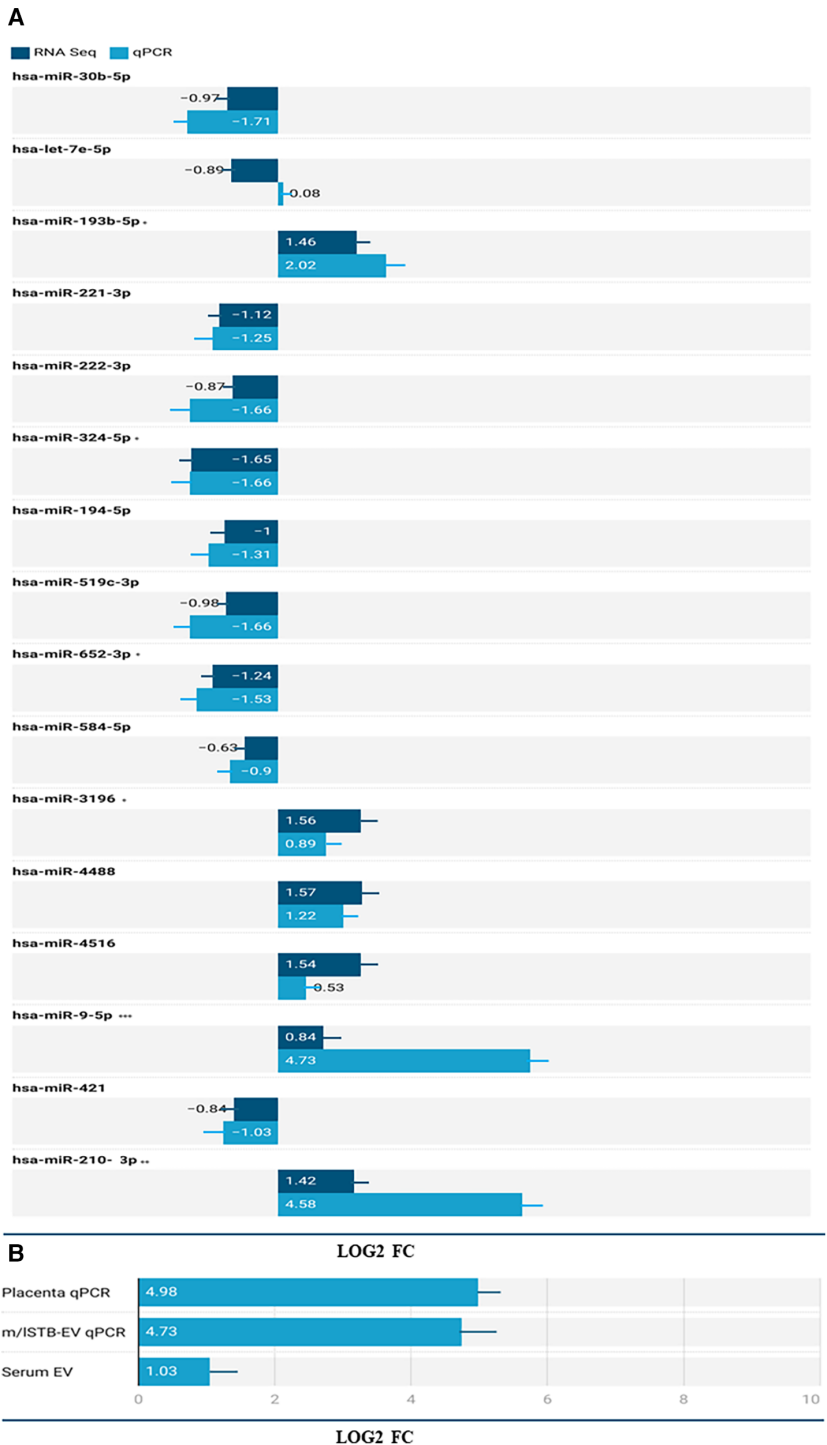
(**A**) Validation of select microRNAs from the RNA Seq dataset in m/lSTB-EVs. The X-axis shows the log2 fold change of the qPCR and RNA Seq data and the Y-axis shows the identity of the select microRNAs. B) differential expression of has-miR-9-5p in the placenta (qPCR), m/lSTB-EVs (qPCR), and serum EV (qPCR) data. *P* < 0.05 is *, *P* < 0.01 is **, *P* < 0.001 is *** and *P* < 0.0001 is ****.

In the placenta, a comparative analysis between preeclampsia (PE) and normal pregnancy revealed significant dysregulation of microRNAs. Specifically, 14 microRNAs were upregulated, while 120 were downregulated (*p*-value < 0.05, as shown in the Supplementary Figure S1). The full list of the placenta data can be found in the Supplementary Material. In comparison between PE and normal pregnancy m/lSTB-EVs, 47 microRNAs were upregulated, and 53 were downregulated (*p*-value of <0.05) (see Supplementary Files for full list). Table 2 shows the top forty DEMs in m/lSTB-EVs in PE. As discussed earlier in the methodology section, due to our interest in m/lSTB-EVs as potential biomarkers, we selected the most differentially expressed microRNAs from the RNA sequencing data. For m/lSTB-EVs (Figure 2A), of the microRNAs tested, hsa-miR-193b-5p (RNA Seq FC = 1.46 qPCR FC = 2.02), hsa-miR-324-5p (RNA Seq FC = −1.65 qPCR FC = −1.66), hsa-miR-652-3p (RNA Seq FC = −1.24 qPCR FC = −1.53), hsa-miR-3196 (RNA Seq FC = 1.59 qPCR FC = 0.89), hsa-miR-9-5p (RNA Seq FC = 0.84 qPCR FC = 4.73), hsa-miR-421 (RNA Seq FC = −0.84 qPCR FC = −1.03), and hsa-miR-210-3p (RNA Seq FC = 1.42 qPCR FC = 4.58) were all significantly differentially expressed. We meticulously scrutinized our validated differentially expressed microRNA repertoire to uncover novel insights. We sought a microRNA with a positive fold change while concurrently exhibiting involvement in angiogenesis—a process intricately linked to the pathology of Preeclampsia. Our investigation led us to hsa-miR-9-5p, a microRNA that remains relatively underexplored within the field despite its pivotal role in angiogenesis. Figure 2B shows that in the placenta (FC = 31.66 SE = 4.98, *P* < 0.001) and maternal serum EVs (FC = 2.04 SE = 0.34, *P* < 0.001), hsa-miR-9-5p is significantly more abundant in PE compared to NP.

**Table 2:** List of differentially expressed microRNAs in medium/large syncytiotrophoblast membrane extracellular vesicles (m/lSTB-EVs).

MicroRNA	Log2 fold change	Adjusted *P* value
hsa-miR-4488	1.57	1.19E-07
hsa-miR-3196	1.56	9.41E-10
hsa-miR-4516	1.54	8.41E-08
hsa-miR-193b-5p	1.46	4.50E-09
hsa-miR-210-3p	1.42	7.68E-08
hsa-miR-27a-5p	1.35	2.09E-06
hsa-miR-3656	1.21	0.0003
p-hsa-miR-113	1.20	4.89E-06
hsa-miR-3960	1.09	0.0002
hsa-miR-6089	1.05	0.0002
hsa-miR-127-3p	1.03	0.0021
p-hsa-miR-317	1.00	0.0014
p-hsa-miR-338	0.90	0.0047
hsa-let-7b-5p	0.87	0.0026
hsa-miR-9-5p	0.85	0.0034
hsa-miR-483-3p	0.85	0.0064
hsa-miR-493-5p	0.83	0.0063
hsa-miR-455-3p	0.82	0.0099
hsa-miR-99a-5p	0.82	0.0087
hsa-miR-370-3p	0.81	0.0106
hsa-miR-10b-5p	0.79	0.0088
hsa-let-7c-5p	0.78	0.0127
hsa-miR-92b-3p	0.78	0.0076
hsa-miR-151a-5p	−0.79	0.0007
hsa-miR-26b-5p	−0.80	0.0001
hsa-miR-519d-3p	−0.80	0.0003
hsa-miR-877-5p	−0.84	0.0088
hsa-miR-421	−0.85	0.0014
hsa-miR-106b-5p	−0.86	0.0026
hsa-miR-93-5p	−0.86	0.0004
hsa-miR-222-3p	−0.87	0.0007
hsa-let-7e-5p	−0.90	8.83E-05
hsa-miR-374a-5p	−0.91	0.0007
hsa-miR-519b-3p	−0.93	0.0002
hsa-miR-30b-5p	−0.97	9.41E-06
hsa-miR-519c-3p	−0.98	0.0002
hsa-miR−194-5p	−1.01	0.0002
hsa-miR-221-3p	−1.12	3.26E-06
hsa-miR-652-3p	−1.24	1.23E-10
hsa-miR-324-5p	−1.65	1.17E-07

### Functional enrichment analysis of differentially expressed microRNAs (DEMs) in preeclampsia

Table 3 presents the functional enrichment analysis results on differentially expressed microRNAs found in both placenta and m/lSTB-EVs. The analysis revealed ten biological processes influenced by upregulated microRNAs, including the neurotrophin TRK receptor signaling pathway, cellular protein modification process, and epidermal growth factor receptor signaling pathway (as detailed in Table 3). Moreover, the KEGG pathway analysis highlighted the significance of ECM-receptor interaction among the upregulated microRNAs (Table 3). In contrast, the downregulated microRNAs exhibited a broader range of shared KEGG pathways, encompassing ECM-receptor interaction, glycosphingolipid biosynthesis (lacto and neolacto series), morphine addiction, TGF-beta signaling pathway, and ErbB signaling pathway. Refer to the Supplementary Material for the complete list of functional enrichment analysis results.

**Table 3:** Table showing the overlapping functional enrichment processes and terms for up and downregulated microRNAs present in both the placenta homogenate and medium/large STB-EVs.

Upregulated microRNAs	
Gene Ontology: Cellular Components	
Placenta and m/lSTB-EVs	Organelle membrane
	Cytosol
	Membrane protein complex
Gene Ontology: Molecular Function	
Placenta and m/lSTB-EVs	Transcription factor binding
	Ion binding
	Enzyme binding
Gene Ontology: Biological Process	
Placenta and m/lSTB-EVs	Transcription
	Biosynthetic process
	Fc-epsilon receptor signaling pathway
	Neurotrophin TRK receptor signaling pathway
	Cellular protein modification process
	Gene expression
	Epidermal growth factor receptor signaling pathway
	Chemical synaptic transmission
	Fibroblast growth factor receptor signaling pathway
	Cell-cell signaling
KEGG Pathways	
Placenta and m/lSTB-EVs	ECM-receptor interaction
Downregulated microRNAs	
Cellular Components	
Placenta and m/lSTB-EVs	Membrane protein complex
	Nucleoplasm
	Cytosol
	Organelle membrane
Molecular Function	
Placenta and m/lSTB-EVs	Transcription factor binding
	Ion binding
	Enzyme binding
	Cytoskeletal protein binding
	RNA binding
KEGG Pathways	
Placenta and m/lSTB-EVs	Fatty acid biosynthesis
	Proteoglycans in cancer
	Mucin type O-Glycan biosynthesis
	Hippo signaling pathway
	Biosynthesis of unsaturated fatty acids
	Glioma
	Axon guidance
	Wnt signaling pathway
	Amoebiasis
	Lysine degradation
	Signaling pathways regulating pluripotency of stem cells
	ECM-receptor interaction
	Glycosphingolipid biosynthesis—lacto and neolacto series
	Morphine addiction
	ErbB signaling pathway
	TGF-beta signaling pathway

### Regulatory MicroRNA and messenger RNA networks in placenta homogenate and medium/large STB-EVs in preeclampsia

**Figure 3:**
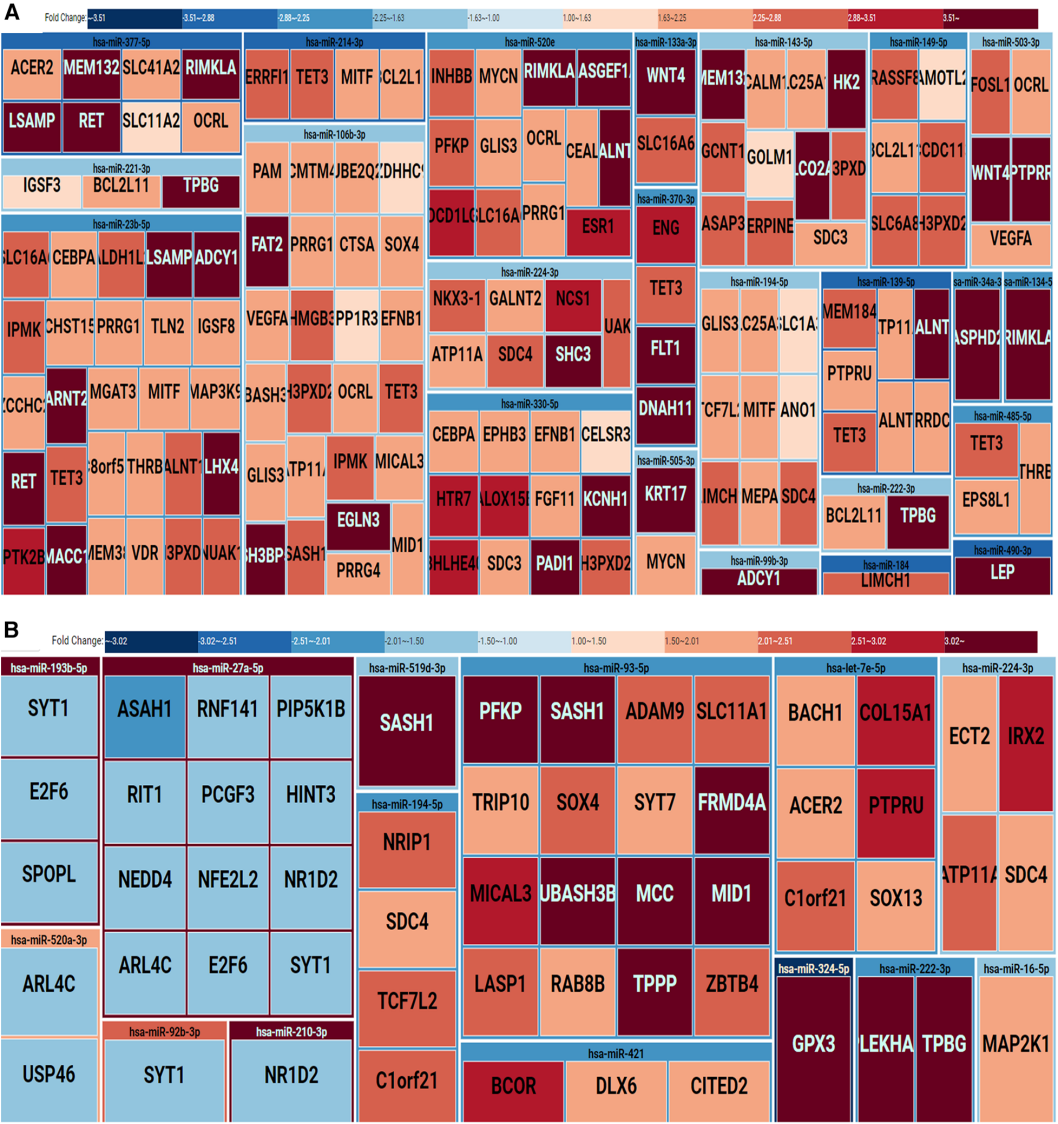
Treemap of microRNA-mRNA regulatory networks in the placenta (**A**) and m/lSTB-EVs (**B**) the treemap is grouped by regulatory microRNAs and their identified mRNA targets. The rectangles are also color-coded: downregulation (blue) or upregulation (red).

To comprehensively assess the potential influence of microRNA modifications on mRNA expression, our investigation systematically cross-referenced the results of microRNA analysis with messenger RNA sequencing data, as outlined in the methods section. Within extracellular vesicles derived from placental and medium/large syncytiotrophoblasts, our analysis revealed 155 and 57 distinct microRNA-mRNA interactions, respectively. Notable instances include the interaction between downregulated hsa-miR-222-3p and upregulated TPBG and PLEKHA2. The visual representation of these interactions is depicted in Figures 3A,B for the placenta and m/lSTB-EVs, respectively. It is essential to highlight that among the microRNAs validated by quantitative polymerase chain reaction (qPCR) in m/lSTB-EVs, only two, specifically hsa-miR-324-5p and hsa-miR-421, exhibited previously confirmed mRNA targets based on other experimental studies, as elaborated in Table 4.

**Table 4:** Previously crosslinking immunoprecipitation (CLIP-Seq) validated microRNA-mRNA targets detected by our correlation analysis.

qPCR validated M/l STB-EV microRNA(s)	mRNA target (s)	Validation method
Hsa-miR-324-5p	GPX3	CLIP-Seq*
	MAFB	
Hsa-miR-421	TBC1D22A	CLIP-Seq*
	ENAH	
	PRKAA2	
	NFIB	
	PLS3	
	JARID2	
	AK4	

* Source of validation is miRTarBase (www.mirtarbase.cuhk.edu.cn), CLIP-Seq, crosslinking and immunoprecipitation sequencing.

## Discussion

Preeclampsia (PE) is a multisystem hypertensive pregnancy disorder with severe maternal and fetal complications. Despite advances in diagnosis, PE remains a diagnostic and therapeutic challenge—these are limited to biomarkers with a high negative predictive value but a low positive predictive value. Our analysis identified an extensive list of microRNAs, including some previously identified PE-related microRNAs, e.g., miR-210, hsa-miR-222-3p, and miR-193b-5p (24–28). Some of our findings were also discordant with previous literature. Zhang et al. (29) identified miR-30b-5p as upregulated in PE placenta and serum, unlike our findings, which revealed a reduction in this microRNA. Likewise, hsa-miR-584 was found to be upregulated in severe PE placenta by Jiang et al., which we did not detect, and Liu et al. found miR- 222-3p to be upregulated in PE plasma and placenta in contrast with our study, which showed a down-regulation in m/lSTB-EVs (not statistically significant) (30, 31). These differences may be due to variations in sample type (plasma vs. serum), spatial placenta microRNA expression variability, sampling region, technical and methodological variances, and analytic differences [RNA Seq (which we performed) compared to microarrays] or might reflect some variability in the phenotype of PE (32). Having identified the most differentially expressed microRNA based on the magnitude of fold change, we confirmed these changes by qPCR. The microRNAs we identified and confirmed as differentially expressed (hsa-miR-324-5p, hsa-miR-3196, hsa-miR-652-3p hsa-miR-9-5p, and hsa-miR-421) have, to the best of our knowledge, not been previously described in Preeclampsia. Hsa-miR-193b-5p (previously described in the placenta, placenta debris) and hsa-miR-210-3p (previously described in the placenta, plasma, serum, plasma exosome), though they have been reported in Preeclampsia have not been previously reported in perfused m/lSTB-EVs (13, 32–37).

Regarding our most important discovery, we found and validated an upregulation of hsa-miR-9-5p in the placenta, medium/large extracellular vesicles, and serum extracellular vesicles of preeclamptic patients. Hsa-miR-9-5p has been studied in the context of angiogenesis in various malignancies, including hepatocellular carcinoma, renal cell cancer, and lung adenocarcinoma (38–40). Still, its role in conditions like Preeclampsia is unknown. In cancer, this microRNA exhibits a dual function. In the case of cervical cancer and human endothelial cells, hsa-miR-9-5p has been observed to promote angiogenesis by targeting SOCS5 and CXCR4, respectively (41, 42).

In contrast, it has a suppressive role in the metastasis of nasopharyngeal carcinoma, gastric cancer melanoma, and brain cancer. Similarly, hsa-miR-9 is a negative regulator of cardiac hypertrophy, suppresses myocardin expression and ameliorates cardiac function, is significantly lower in hypertensive patients compared to healthy individuals, positively correlates with left ventricular mass, and may serve as a potential marker or indicator of target organ damage (43, 44). It could be that hsa-miR-9-5p is elevated in Preeclampsia as part of an endogenous repair mechanism to counteract the anti-angiogenic environment in the condition. Alternatively, hsa-miR-9-5p might inherently function as an anti-angiogenic factor in Preeclampsia and a marker of end-organ damage observed in cancers. These possibilities require further investigation through additional studies. It would be interesting to explore the role of circulating hsa-miR-9-5p in Preeclampsia.

Upon identifying a suite of differentially expressed microRNA, we embarked on functional enrichment analysis, which confirmed biological processes known to be abnormal in Preeclampsia, such as abnormal cellular protein modification process, neurotrophin TRK receptor signaling pathway (45), epidermal growth factor receptor signaling pathway (46), cell motility (47), stress response (48), Fc-epsilon receptor signaling pathway (49) and TGF-beta signaling pathway (50). The pathogenesis of Preeclampsia is characterized by intricate molecular mechanisms involving the dysregulation of multiple signaling pathways. A critical pathway in placental function is the unfolded protein response (UPR), triggered by stressors such as hypoxia and nutrient deprivation. Numerous studies consistently highlight the significant role of UPR in placental function. In individuals with Preeclampsia, excessive activation of UPR in the placenta is observed, leading to the endoplasmic reticulum (ER) stress (51–53). This activation induces the NOD-like receptor pyrin-containing 3 (NLRP3) inflammasome, resulting in pyroptosis in trophoblasts (53).

Additionally, UPR is linked to lysosomal homeostasis disruptions and autophagic flux blockade initiation in human trophoblasts, further underscoring its involvement in preeclampsia pathophysiology (54). Distinct activation patterns of UPR in early- and late-onset Preeclampsia, its role in placental oxidative phosphorylation, and its contribution to disease heterogeneity highlight its significance (55, 56). A comprehensive understanding of these mechanisms, including the roles of microRNAs, can potentially inform targeted therapeutic interventions.

We also explored mRNA targets associated with differentially expressed microRNAs to illuminate the underlying regulatory mechanisms. Through this analysis, we sought to discern the extent to which previously identified messenger RNA changes could be attributed to alterations in the microRNA profile (16). Specific interactions, particularly within the m/lSTB-EVs, were corroborated by others using CHIP-Seq validation methods (Table 4). Remarkably, 98% of the identified microRNA-mRNA interactions demonstrated a pattern of downregulated microRNAs coupled with upregulated target mRNAs, aligning with expected outcomes. The remaining 2% lacked a microRNA-based explanation for the observed upregulated mRNA transcripts, suggesting the presence of alternative mRNA regulatory mechanisms beyond microRNAs. These mechanisms encompass RNA binding proteins, transcription factors, nonsense-mediated mRNA decay (NMD), and other small RNAs (57, 58). Encouragingly, essential differentially expressed genes implicated in Preeclampsia, such as FLT-1, Endoglin, and ADAMTS, were predicted targets of the differentially expressed microRNAs in our study (59, 60). Two regulatory microRNA networks previously validated (Table 4) detected in our study were hsa-miR-421 and hsa-324-5p. The investigation into the role hsa-miR-421 in Preeclampsia has garnered considerable attention in recent research. Notably, studies have delved into the differential expression hsa-miR-421, examining its implications in HIV-associated Preeclampsia and its associations with COVID-19 (61).

Moreover, a distinct facet of this inquiry has unveiled that circ_0007121 plays a pivotal role in enhancing trophoblastic cell proliferation, migration, and invasion. This effect is achieved through the intricate regulation of the hsa-miR-421/ZEB1 axis in the context of Preeclampsia (62). In a parallel line of investigation, miR-1246 has emerged as a noteworthy player, exhibiting significant decreases in expression within the placentas of women afflicted by severe Preeclampsia. Intriguingly, this alteration coincides with elevated levels of AXIN2, GSK3β, and JARID2 compared to normotensive subjects (63). Additionally, the potential therapeutic significance of JARID2 in addressing Preeclampsia has been proposed. This proposition is grounded in the observed inhibitory effects of JARID2 on the viability and migration of placental trophoblast cells, as elucidated in previous research (64).

Similarly, hsa-miR-324-5p's regulatory functions extend to cell proliferation, invasion, apoptosis, hedgehog signaling, adipocyte differentiation, and lipid droplet accumulation, all pertinent to its potential role in Preeclampsia (57, 58). In the specific context of Preeclampsia, the significance of GPX3, a selenoprotein with antioxidant properties, emerges. Existing studies indicate reduced GPX3 expression in Preeclampsia, with selenium deficiency implicated in pregnancy disorders, including Preeclampsia (65, 66). Furthermore, differential expression of GPX3 in the placenta during normal and preeclamptic pregnancies underscores its potential involvement in the pathophysiology of Preeclampsia. This prediction correlated with the observed upregulation of mRNA associated with the downregulation of microRNA, further supporting the relevance of these genes in the context of Preeclampsia.

Our study has numerous positives, particularly our ability to identify potential STB-EV microRNA biomarkers, which might help in the early diagnosis of PE. However, when interpreting our results, it is crucial to note that the control population used for this study was not gestationally age-matched with PE patients (it is impossible to obtain the ideal control for PE patients), particularly the early onset phenotype (<34 weeks). Therefore, we used term (37 to 41 + 6 weeks) controls. However, we made up for this by exploring the differential abundance of hsa-miR-9-5p in gestational age-matched maternal serum samples. In addition, our sample size, 6 for STB-EVs and 8 for serum EVs, is still relatively small.

## Conclusion

In conclusion, we have identified new STB-EV-carried microRNAs, particularly hsa-miR-9-5p, that may be diagnostic in PE. We have also identified some vital pathological pathways that are dysregulated in preeclampsia and might serve as mechanistic or therapeutic targets for PE. Finally, we correlated the results of this microRNA analysis with the results of our previously published mRNA analysis to identify a potential microRNA-mRNA regulatory network, which may form the subject of future studies and help to better understand the pathogenesis of Preeclampsia.

## Data Availability

The datasets presented in this study can be found in online repositories. The names of the repository/repositories and accession number(s) can be found below: https://www.ncbi.nlm.nih.gov/, GSE190972 http://www.EVTRACK.org, EV210382.
