# Supplementary material for: MicroRNA analysis of medium/large placenta extracellular vesicles in normal and preeclampsia pregnancies
Source: Front Cardiovasc Med. 2024 Apr 2;11:1371168. doi: 10.3389/fcvm.2024.1371168 (PMC11018924; doi:10.3389/fcvm.2024.1371168)
Supplement: Supplementary file 1 [file Datasheet1.pdf]

Supplementary Material

Supplementary Methods

Human endothelial umbilical vein cell (HUVEC) lysates as a positive control for western blots

Human umbilical vein endothelial cells (HUVEC) were isolated from the human umbilical cord by collagenase digestion of the interior of the umbilical vein. Briefly, placentas were collected from normal pregnant women undergoing elective cesarean section and processed within 10 minutes. The umbilical cord was inspected, and if healthy, the cord was clamped and separated from the placenta. Next, about ten to fifteen centimeters of the cord with no evidence of bleeding or trauma was set aside for HUVEC isolation. The umbilical vein from the cord was identified. The vein was flushed with 20 ml Hanks' solution till the effluent was clear to ensure the patency of the umbilical vein and to get rid of red blood cells. Air was injected twice to get rid of the Hanks solution. The lower end of the umbilical cord was clamped with artery forceps, and the upper end of the vein was infused with 5 ml of 1X collagenase type IA (1mg/ml) (Sigma, UK). The cord was then incubated at 37°C for 15 minutes in 5% CO2 for maximal digestion of the extracellular matrix and the detachment of endothelial cells. During incubation, the cord was intermittently massaged every five minutes. After fifteen minutes, the contents of the umbilical vein were emptied into a ten-milliliter Falcon tube, followed by flushing the cord twice with ten milliliters of complete endothelial cell medium (Life Technologies). The cell suspension was pelleted at room temperature at 500 g for 5 minutes. The pellet was then re-suspended with 2 milliliters of complete endothelial cell medium, seeded equally into two 1% gelatin-coated T25 cell culture flasks (NUNC^{TM}), and topped with 8 milliliters of complete endothelial cell medium. Cells were left to settle for 2 hours in 5% CO2 and 21% O2, after which the media was changed to eliminate contaminating red blood cells, then every two days until the cells were 89-90% confluent. HUVECs were passaged to make more cells, processed as cell lysates, or stored in liquid nitrogen for future use. Trypan blue, a dye impermeable to healthy cells but not dead or dying cells, was used to check for viability by direct visual observation under the microscope. Equal volumes of cell suspension and trypan blue (0.4% w/v solution) were mixed and loaded on a hemocytometer (superior Marienfled). After verifying the viability of cells, 2 X 10^6 cells were resuspended in 500 microlitres of fPBS and lysed with RIPAs buffer (Thermo Fisher^{TM}) buffer using the standard protocol to generate cell lysates.

Bicinchoninic Acid (BCA) protein assay

Fresh lysed HUVEC cells were assayed for their protein content using the bicinchoninic acid protein assay kit (BCA) (Pierce). Briefly, bovine serum albumin (BSA) stock (1 mg/ml) was diluted in filtered PBS to give rise to protein concentration standards 1,0.5,0.25,0.125,0.625 mg/ml. HUVEC lysates were diluted in filtered PBS (1: 8 ratio). Twelve and a half microlitres of BSA standards and samples were added to a 96-well plate, followed by 100 microlitres of BCA reagent mix (50:1 ratio of BCA solution and copper II sulfate solution, respectively). Proteins reduce Cu^{2+} to Cu^{+} in an alkaline solution (the biuret reaction), resulting in a purple color, read by a spectrophotometer to extrapolate protein concentration based on a standard curve. The 96-well plate was covered in foil and put in the shaker for 5 minutes before 30 minutes incubation at 37°C. Reactions were performed in duplicates. Absorbance was read at 562 nm using a FLUOStar OPTIMA (BMG) plate reader.

Enrichment of STB-EVs by placental dual-lobe perfusion and serial ultracentrifugation

We located a suitable cotyledon, ensuring it was free of calcifications, ischemia, or rupture. Subsequently, we cannulated a placental artery and vein, perfusing the placenta for three hours at a flow rate of 4-5 ml/min to obtain the placenta perfusate. This perfusate underwent two centrifugation steps at 1,500 g for ten minutes at 4°C using a Beckman Coulter Avanti J-20XP centrifuge equipped with a Beckman Coulter JS-5.3 swing-out rotor to remove cell debris. After pooling the resulting supernatant, it underwent a spin at 10,000 g (10K) in a swing bucket centrifuge (Beckman L80 ultracentrifuge and Sorvall TST28.39 swing-out rotor) at 4°C for 30 minutes. The 10K STB-EV pellet was washed with filtered phosphate-buffered saline (fPBS), followed by resuspension of the 10K STB-EV pellets in fPBS. An aliquot of the resuspended pellets was analyzed to identify and characterize STB-EVs. The remaining aliquots were utilized to achieve a protein concentration of approximately 2-5 µg/µl (measured using a Pierce Bicinchoninic Acid (BCA) protein assay) and promptly stored at -80°C. This prepared stock was used for subsequent analyses.

Transmission electron microscopy

10K STB-EV pellets were mixed with fPBS to create a 10K STB-EV solution with concentrations ranging from 0.1 to 0.3 µg/µl. For two minutes, a ten-microliter aliquot of the STB-EV pellet solution was applied to freshly glow-discharged carbon formvar 300 mesh copper grids. The grids were then blotted with filter paper, stained with 2% uranyl acetate for ten seconds, and left to air-dry. Negative staining was employed on the 10K STB-EV pellets on the grid to enhance the contrast between the 10K STB-EV pellets and the background. The grids were imaged using an FEI Tecnai 12 transmission electron microscope at 120 kV, equipped with a Gatan OneView CMOS camera.

Flow Cytometry

All sample analyses were conducted by a BD LSRII flow cytometer (BD Biosciences) equipped with blue, violet, and red lasers. Daily quality control (QC) involved using CS&T beads (BD Biosciences). The photomultiplier tube (PMT) voltage, determined during the CS&T run, was applied to all fluorescent detectors except for side scatter (SSC), which was determined by Apogee Mix (1493, Apogee Flow System, UK). The SSC PMT voltage triggering 0.59 µm silica beads and above was applied to all 10K STB-EV pellets and analyzed. An SSC threshold of 200 was applied to eliminate background noise below 0.59 µm silica beads. A flow rate of 10 µl/min was maintained using TruCount beads (BD Science). For sample staining, 90 ml of the 10K STB-EV pellet was incubated with ten ml of Fc receptor blocker (Miltenyi, UK) for 10 minutes at 4°C and then stained with phycoerythrin (PE)-conjugated PLAP (for syncytiotrophoblast origin) for ten minutes at room temperature in the dark. Stained samples were transferred to an Ultrafree 0.2 µm filter unit (Millipore) and centrifuged at 800 g for three minutes to remove unbound antibodies and EVs smaller than the filter pore size. Ninety microliters of fPBS were used to recover 10K STB-EVs retained on the filter membrane. Recovered 10K STB-EVs were further stained with BODIPY FL N-(2-aminoethyl)-maleimide [505/513 nm] (Molecular Probes) at a final concentration of 0.5 nM in the dark at room temperature for ten minutes before samples were diluted to 500 ml and analyzed on the flow cytometer to check for events rate.

When necessary, dilutions were made to achieve an events rate of ≤ 400 counts/second and to reduce swarming. 10K STB-EV pellets were analyzed at 10 µl/minute for 10 minutes, and 100 µl diluted samples were analyzed for each. Fluorescence minus one (FMO-1) for each fluorochrome and stained samples reacquired after 2% Nonidet P-40 (NP-40) (Sigma) treatment were used as controls. Data and figures were generated with FlowJo software version 10 (Tree Star Inc., Ashland, OR).

Nanoparticle Tracking Analysis

We conducted further characterization of the 10K pellets using nanoparticle tracking analysis (NTA) with a NanoSight NS500 instrument equipped with a 405 nm laser (Malvern UK), sCMOS camera, and NTA software version 2.3, Build 0033 (Malvern UK). Before sample analysis, we verified instrument performance using silica 100 nm microspheres (Polysciences, Inc.). For analysis, the 10K STB-EV pellets were individually diluted in fPBS to a range of 1/100,000. The samples were automatically injected into the sample chamber using a 1 ml syringe with the following script for EV measurements: prime, delay 5, capture 60, repeat 4. Images of the analyzed samples were captured on the camera at level 12 (Camera shutter speed: 15 milliseconds and Camera gain: 350). NTA post-acquisition settings were optimized and maintained consistently across samples. Each video recording was analyzed to deduce the size and concentration profile of STB-EVs.

Bioinformatic analysis of microRNA sequences

The bioinformatics analysis for micro-RNA was conducted using Oasis 2.0 (https://oasis.ims.bio/small_rna_classification.php), a bioinformatic web tool known for its speed, reliability, and accessibility. Initially, the fastq files underwent compression using the Oasis compressor to facilitate subsequent analysis. The compressed files were then uploaded to the Oasis 2.0 web tool. Quality control (QC) metrics were obtained using FastQC (v.0.11.2) both before and after the trimming of adapters/barcodes [trimmomatic (v.0.32)], along with length filtering set at a minimum of 15 and a maximum of 50 nucleotides. Acceptable mismatches were defined as 5% of the read length. The reads were aligned against the human reference genome, micro RNAome, and sRNAome (piwi-interacting RNA, small interfering RNA, small nuclear RNA, and small nucleolar RNA) stored in the Oasis database using STAR (2.4.1d). Unmapped reads from this step underwent realignment to the human reference genome (Homo Sapiens hg38) for the prediction and archival of novel micro RNAs in the Oasis-Db, utilizing Bowtie (v1.0.0). Subsequently, unmapped reads from this second step were mapped to bacterial, archaeal, and viral genomes using Kraken (v.0.15.5-beta) to identify and characterize infectious contaminants.

For the detection of potentially orthologous or cross-species microRNA reads that could not be classified as human or infectious contaminants in any of the three prior steps, realignment was carried out against all non-human micro RNAs in miRbase 208 using STAR (v.2.4.1d). Any remaining unmapped reads after these four simultaneous steps were subsequently discarded. Detailed information regarding the default parameter settings for OASIS-db can be found in the accompanying documentation. Gene expression values were quantified using STAR quant mode, and exploratory analysis of the results, including visualization, was conducted on Oasis. A gene count table was generated from each gene count.

Differential expression analysis of microRNA data

The DESeq2 package (v.1.32.0 in R v.4.0.5) generated a list of differentially expressed small RNAs between normal and preeclampsia samples, following the standard protocol and accompanying DESeq's tutorial. P values were adjusted for multiple tests using the Benjamin-Hochberg method, and these corrected p values were reported as a false discovery rate, with significance defined as less than 0.05. In DESeq2, the gene count data were prefiltered to eliminate genes with fewer than five reads across all samples. The data were then visually explored using principal component analysis and Pearson correlation heat maps. Samples were identified as outliers if clustered away from others and had a correlation score of less than 0.8. Differentially expressed small RNAs were screened with the criteria of a false discovery rate (FDR) <0.05 and |log2(Fold change) | ≥ 0.75.

Complementary cDNA synthesis for microRNA synthesis

Complementary DNA (cDNA) was synthesized using the TaqMan™ Advanced miRNA cDNA Synthesis Kit (A28007). An equivalent quantity of RNA samples was set aside before initiating the reverse transcription of RNA samples. To each aliquot, an equal amount of cel-mir-39 (Integrated DNA Technologies, 100 nmoles RNA Oligo) spike-in was added (1 µl per sample from a 0.25 nanomolar working solution). All reactions were conducted on ice, following the manufacturer's protocol.

The reverse transcription phase consisted of four stages: 1) polyA tailing, 2) adaptor ligation, 3) reverse transcription, and 4) miR-amplification reaction. Unlike mRNAs, MicroRNAs, short nucleotide sequences without a polyA tail, presented a challenge in reverse transcription due to their short sequences. To overcome this, a polyA tail was artificially added to facilitate reverse transcription. Each reaction was carried out in a G-storm™ (G-storm, UK) thermocycler following the manufacturer's provided settings.

After reverse transcription, ten µl of reverse-transcribed samples underwent preamplification. Unpreamplified samples were aliquoted and stored at -80oC for future use. The preamplified samples were diluted at 1:5 with 0.1X TE buffer for subsequent qPCR reactions. In cases where qPCR could not be performed on the same day due to the requirement for preamplification, aliquots were stored in the refrigerator (for < 2 days) or at -20°C (for less than a month).

Quantitative polymerase chain reaction (qPCR)

The selected differentially expressed micro RNAs were validated through quantitative polymerase chain reaction using the hydrolysis probe-based Taqman® gene expression assay (Applied Biosystems, USA). The Taqman® technology followed a well-described workflow summarized in Figure 10. A qPCR master mix was prepared, consisting of 1 µl of 20X Taqman gene expression assay, 10 µl of 2X Taqman® gene expression master mix, 7 µl of RNAse-free water. Eighteen microliters of this master mix were added to 2 µl of preamplified cDNA samples in a 96-well reaction plate (MicroAmp™ Optical 96-Well Reaction Plate), sealed with a transparent qPCR film (MicroAmp™ Optical Adhesive Film) to prevent sample loss by evaporation.

Forty cycles per reaction were carried out using the Quantstudio™ 3 real-time PCR System (ThermoFisher), and all tested micro RNAs were detected before the 30th cycle. The qPCR settings were as follows: hold at 50°C for 2 minutes, hold at 95°C for 20 seconds, followed by forty cycles of

95°C for 1 second and 60°C for 20 seconds, acquiring to the FAM^{TM} Channel. Cq values were automatically generated by the Quantstudio Design and Analysis desktop software (ThermoFisher). Raw data obtained from the software were exported as an Excel spreadsheet, and subsequent analysis was performed in Microsoft Excel. As stated earlier, the relevant reference microRNAs were used to normalize all qPCR data. The selection of hsa-miR-30d-5p was based on the sequencing data, demonstrating absent differential expression (FC < 0.001) between PE and normal, along with a low standard error (LSE).

An independent qPCR experiment was conducted to validate the stability of hsa-30d-5p. qPCR data and fold change (FC) were calculated using the $2^{-\Delta\Delta Ct}$ method. Statistical testing was performed on the ΔCt values using a one-tailed Student t-test, with significance set at $p < 0.05$. Data visualization was accomplished with GraphPad Prism software (version 9), expressing fold change and denoting standard error as error bars: supplementary Table 1 lists all microRNA expression assays and their corresponding assay IDs in this study.

Supplementary Table 1. Quantitative polymerase chain reaction (PCR) primers, assay ID and amplicon length.

Micro RNA	Assay ID
Hsa-miR-4516	478303_mir
Hsa-miR-193b-5p	478742_mir
Hsa-miR-3196	483159_mir
Hsa-miR-4488	478906_mir
Hsa-miR-652-3p	478189_mir
Hsa-miR-584-5p	478167_mir
Hsa-miR-194-5p	477956_mir
Hsa-miR-519c-3p	479495_mir
Hsa-miR-324-5p	483066_mir
Hsa-miR-30b-5p	478007_mir
Hsa-miR-221-3p	477981_mir
Hsa-miR-191-5p	477952_mir
Hsa-miR-222-3p	477982_mir
Hsa-miR-30d-3p	479362_mir
Hsa-miR-9-5p	478214_mir
Hsa-let-7e-5p	478579_mir
Hsa-miR-210-3p	477970_mir

Supplementary Table 2: Major Resources Table for Western Blot

**Target antigen**	**Vendor or Source**	**Catalog #**	**Working concentration**	**Dilution**
PLAP	In house antibody		1.6 µg/µl	1/1000
CD63	Santa Cruz Biotechnology	sc-365604<br>RRID: AB_10847220	200 µg/µl	1/1000
Cytochrome C	Santa Cruz Biotechnology	sc-13560<br>RRID: AB_627383	200 µg/µl	1/500
Mouse Immunoglobulins	Dako UK Ltd	P044701<br>RRID: AB_2617137		1/2000
Rabbit Immunoglobulins	Dako UK Ltd	P044801<br>RRID: AB_2617138		1/2000

Supplementary Table 3: Major Resources Table for Flow Cytometry Resources

Markers	Fluorochromes	Clone	CAT	Isotype	Dilution / Concentration
PLAP	PE	Mouse mAb	N/A	IgG1	0.2mg/ml
IgG1 isotype control	Pacific blue	MOPC-21	400131<br>RRID: AB_2923473	IgG1	1 in 200
bioM	FITC	NA	NA	NA	0.5-1nM

Supplementary Results

General characteristics of the Serum extracellular vesicles (EV) samples used for validation

Supplementary Table 4 presents a comprehensive comparative analysis between normal pregnancies and those affected by preeclampsia, examining various parameters, including sample size, maternal age, body mass index (BMI), blood pressure metrics, proteinuria manifestation, gestational age at delivery and sample collection, incidence of intrauterine growth restriction (IUGR), and distribution of newborn genders. Notably, the two cohorts demonstrated statistically significant disparities across several metrics. Preeclamptic pregnancies exhibited markedly elevated systolic and diastolic blood pressure readings, as well as a notable prevalence of proteinuria, relative to their normal counterparts ($p < 0.001$). Furthermore, the gestational age at delivery was significantly reduced in preeclampsia cases ($p < 0.001$). Additionally, a substantially elevated occurrence of intrauterine growth restriction (IUGR) was identified within the preeclampsia cohort ($p < 0.001$), indicating compromised fetal development in these instances. However, parameters such as maternal age, BMI, gestational age at sample collection, and the distribution of newborn genders did not demonstrate statistically significant discrepancies between the two groups.

Supplementary Table 4. General characteristics of the Serum EV samples used for validation.

Characteristics	Normal Pregnancy	Preeclampsia	P Value
Sample size	8	8	
Maternal age years (mean (SD))	30 (5.66)	35.63 (6.35)	0.08
Body mass index kg/m^{-2} (mean (SD))	33.70 (13.78)	28.14 (12.28)	0.320
**Systolic blood pressure mmHg (mean (SD))**	**127.75 (8.29)**	**170.00 (32.42)**	**0.001**
**Diastolic blood pressure mmHg (mean (SD))**	**77.63 (4.47)**	**111.25 (24.27)**	**0.001**
**Proteinuria plus(es) (mean (SD))**	**0**	**2.75 (1.51)**	**<0.001**
**Gestational age at delivery in weeks (mean (SD))**	**38.25 (1.28)**	**33.13 (3.65)**	**<0.001**
Gestational age at sample collection in weeks (mean (SD))	30.25 (2.60)	30.13 (2.20)	0.926
**Intrauterine growth restriction (IUGR) = Yes (%)$^{-}$**	**0 (0)**	**7 (87.50)**	**<0.001**
Male new-born gender (%)	4 (50.00)	4 (50.00)	1

MicroRNAs (DEMs) expression in the Placenta and medium/large STB-EVs.

MicroRNA expression in placental tissue and medium/large syncytiotrophoblast extracellular vesicles (STB-EVs) was examined to identify potential enrichment patterns. Using a methodical approach, we identified microRNAs enriched in placental tissue or medium/large STB-EVs by subsetting microRNA expression prevalent in 80% of the samples. Selective enrichment of microRNAs was determined through their exclusive presence in the placenta, medium/large STB-EVs, or both sample types. Of the identified microRNAs, 868 were present in more than 80% of placental samples, while 458 were prevalent in medium/large STB-EVs. Notably, 422 microRNAs were exclusively found in the placenta, whereas only 12 were uniquely present in the medium/large STB-EVs, with 446 microRNAs common to both sample types. The 12 microRNAs uniquely present in medium/large STB-EVs included hsa-miR-320c, hsa-miR-561-3p, hsa-miR-217, hsa-miR-3065-3p, hsa-miR-3925-5p, hsa-miR-3927-3p, hsa-miR-4451, hsa-miR-4652-5p, hsa-miR-766-5p, hsa-miR-138-1-3p, hsa-miR-4787-5p, and hsa-miR-548w.

Further analysis compared the microRNA differential expression profiles between placental tissues from pregnancies complicated by preeclampsia (PE) and those from normal pregnancies (NP). This analysis revealed 14 upregulated and 120 downregulated microRNAs in preeclampsia, exhibiting statistical significance with a p-value of less than 0.05. Supplementary Figure 1 provides a graphical depiction of the differentially expressed microRNAs (DEMs) observed in the placenta under preeclampsia. The supplementary Excel files show a comprehensive list of identified microRNAs in the placental sample and their fold change.

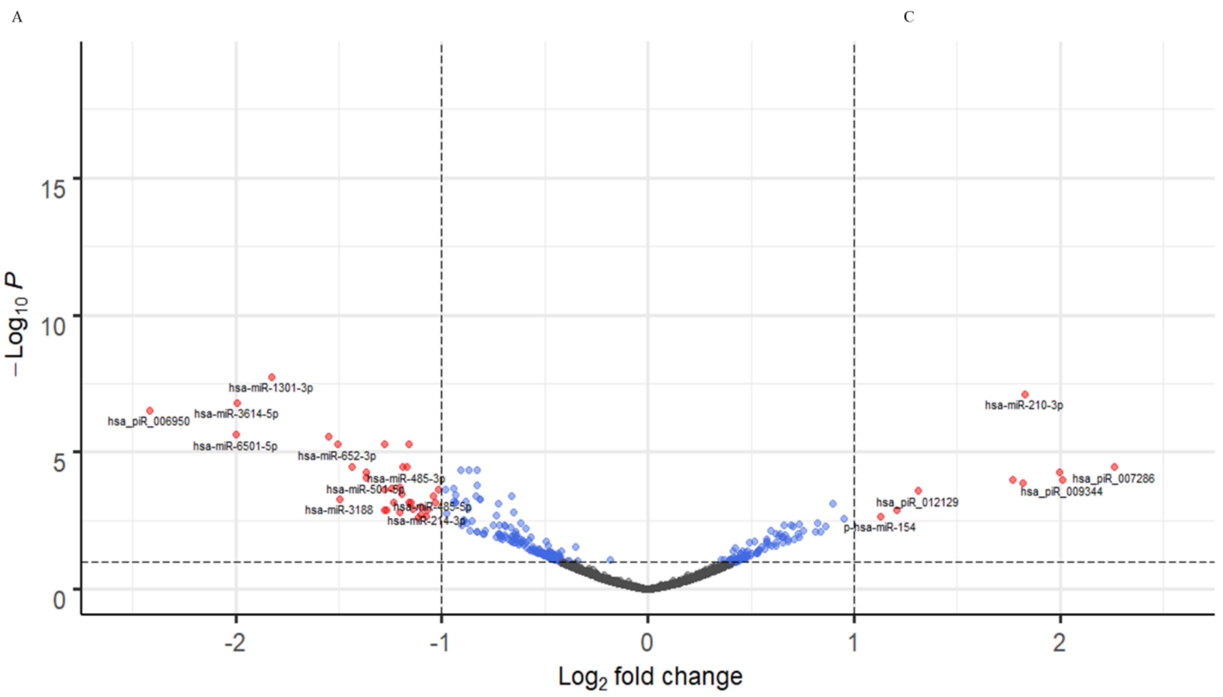

Supplementary Figure 1. Volcano plot showing the differentially expressed small RNAs in the placenta.
